# Regulation of hepatic TRB3/Akt interaction induced by physical exercise and its effect on the hepatic glucose production in an insulin resistance state

**DOI:** 10.1186/s13098-015-0064-x

**Published:** 2015-08-18

**Authors:** Rodolfo Marinho, Rania A. Mekary, Vitor Rosetto Muñoz, Ricardo José Gomes, José Rodrigo Pauli, Leandro Pereira de Moura

**Affiliations:** São Paulo State University, UNESP, Rio Claro, SP Brazil; Faculty of Applied Science, University of Campinas (UNICAMP), Rua Pedro Zaccaria, 1300, Jardim Santa Luzia, Limeira, SP Brazil; Department of Social and Administrative Sciences, MCPHS University, Boston, MA USA; Department of Nutrition, Harvard T. Chan School of Public Health, Boston, MA USA; Department of Biosciences, São Paulo Federal University (UNIFESP), Santos, SP Brazil

**Keywords:** Physical exercise, TRB3, Insulin resistance, Liver, Hepatic glucose production

## Abstract

To maintain euglycemia in healthy organisms, hepatic glucose production is increased during fasting and decreased during the postprandial period. This whole process is supported by insulin levels. These responses are associated with the insulin signaling pathway and the reduction in the activity of key gluconeogenic enzymes, resulting in a decrease of hepatic glucose production. On the other hand, defects in the liver insulin signaling pathway might promote inadequate suppression of gluconeogenesis, leading to hyperglycemia during fasting and after meals. The hepatocyte nuclear factor 4, the transcription cofactor PGC1-α, and the transcription factor Foxo1 have fundamental roles in regulating gluconeogenesis. The loss of insulin action is associated with the production of pro-inflammatory biomolecules in obesity conditions. Among the molecular mechanisms involved, we emphasize in this review the participation of TRB3 protein (*a mammalian homolog of Drosophila tribbles)*, which is able to inhibit Akt activity and, thereby, maintain Foxo1 activity in the nucleus of hepatocytes, inducing hyperglycemia. In contrast, physical exercise has been shown as an important tool to reduce insulin resistance in the liver by reducing the inflammatory process, including the inhibition of TRB3 and, therefore, suppressing gluconeogenesis. The understanding of these new mechanisms by which physical exercise regulates glucose homeostasis has critical importance for the understanding and prevention of diabetes.

## Background

Diabetes *mellitus* type 2 (DM2) is highly prevalent in modern society and is closely related to obesity. Its genesis is linked to insulin resistance (pre-diabetes condition) and there seems to be a triad of recurrent and intricate phenomena capable of contributing to the patient’s hyperglycemic condition with DM2. These are the following: (1) increased hepatic glucose production (via marked increase of glycogenolysis and gluconeogenesis molecules); (2) decreased insulin secretion and activity; and (3) reduced usage and storage of glucose in the body [1, 2].

The normal activity of glucagon and insulin signaling pathway in the liver is important for the control of hepatic glucose production. On one hand, glucagon promotes gluconeogenesis through two pathways: The first is via activating PGC-1α that will interact with FoxO1 and then increase the synthesis of phosphoenolpyruvate carboxykinase (PEPCK) and G6Pase, consequently increasing the gluconeogenesis in the liver [3]. The second way is via increasing Inositol 1,4,5-triphosphate (IP3), which acts on the calcium-releasing channels of the endoplasmic reticulum (ER) by releasing calcium (Ca) to the cytosol. In the cytosol, the Ca will act as a second messenger activating the cytoplasmic calcium-sensitive kinase and the calcium/calmodulin dependent-protein kinase II (CaMKII). In turn, CaMKII will activate MAPK p38α that will phosphorylate FoxO1 at Ser295 and 475, as well as Ser246; the phosphorylated FoxO1 will then enter the cell nucleus and consequently bind to PGC-1α to induce target genes that are responsible for the transcription of genes responsible for glycogenolysis and gluconeogenesis such as G6Pase and Phosphoenolpyruvate carboxykinase 1 (PCK1) in the liver [4–6]. On the other hand, insulin activation promotes a reduction in the expression and activity of the gluconeogenic enzymes, such as PEPCK and G6Pase, which results in a decreased hepatic glucose production [7–9]. In contrast, when hepatic insulin signaling is compromised, an inadequate suppression of gluconeogenic pathways will ensue, which will lead to high levels of glucose and insulin in postprandial and fasting states [7, 10].

The regular practice of physical exercise is a non-pharmacological strategy recognized and indicated for the prevention and treatment of diabetes. It was demonstrated in a previous study that lifestyle changes, including the practice of regular physical exercise and nutritional education, had higher protective effect against the development of diabetes when compared to the sole use of metformin [11]. This scientific evidence indicates that exercise is indeed an important strategy in preventing insulin resistance and should be used to circumvent the development of diabetes.

However, it is still not clear how physical exercise promotes a reduction in hyperglycemia. Furthermore, it is observed in the literature that most studies used skeletal muscle changes as their primary outcome [12–15]. In fact, many of the beneficial effects of physical exercise occur in other tissues which might not be directly involved in muscle contraction. This review was conducted to show how physical exercise could influence hepatic glucose production (with an emphasis on gluconeogenesis) and contribute to the maintenance of glucose homeostasis in a state of insulin resistance. In particular, this review elucidated the effects of physical exercise on TRB3 levels by minimizing its adverse effects on the insulin signaling pathway and the subsequent increase in gluconeogenesis in the liver.

## Obesity and insulin resistance: the role of TRB3 protein

The relationship between obesity and insulin resistance has been intensively investigated and it is known that many biomolecules from the adipocytes are capable of inducing insulin resistance through different mechanisms. A recent study has shown that some types of fatty acids originating from the diet, especially saturated fatty acids, are able to bind to -and thus activate- membrane proteins referred to as *toll like receptor* 4 (TLR4) [16]. TLR4 are mediators of the immunological innate response and their activation promotes negative consequences on the insulin pathway, such as the activation of serine kinases [16, 17]. More specifically, the activation of TLR4 induces the activation of IKKβ (Ikappakinase beta) and JNK (c-jun N-terminal kinase), resulting in the phosphorylation of the insulin receptor (IR) and the insulin receptor substrates 1 and 2 (IRS-1/2) in a serin residue (instead of treonin), which leads to a decreased insulin signal in many tissues, including the liver [16].

TLR4 can also activate the complex called IKK/IKB/NFKB. When active, IKKβ promotes the phosphorylation of IKB, culminating in the dissociation of the IKB/NFKB complex. Once free in the cell, NFKB is able to translocate to the nucleus and promote the transcription of new inflammatory cytokines, such as TNF-α. This pro-inflammatory protein leads to an increased inflammatory level -also called a chronic low-grade inflammation- which seems to be strictly associated with obesity and insulin signaling impairment [16–18].

Notably, TLR4 is associated with TRB3 synthesis through the development of the ER stress [19]. The ER dysfunction arises as a consequence of TLR4 activation and can be modulated by anti-inflammatory cytokines [20]. Yao et al. observed that the activation of TLR4 by modified low-density lipoproteins is essential to induce ER stress in the macrophages [21]. Pierre et al. [22] demonstrated a strong relationship between TLR4 and ER stress; the authors showed that knockout mice for TLR-4 in the liver, fat, and muscle tissues and that were fed a high fat diet did not develop ER stress. TLR4/2 activation in the macrophages activates IRE1/XBP1s and contributes to proinflammatory cytokine production. Moreover, Koh and colleagues [23] reported that high fat diet, obesity, and T2DM induce stress reticulum and in response to this condition, TRB3 levels increase in muscle cells. Based on these findings, the authors hypothesized that impairments in insulin signaling could result from the increase in TRB3 levels. To confirm this hypothesis, the authors first overexpressed TRB3 and observed impairments in insulin signaling; on the other hand, when the transcription of TRB3 was silenced, the reverse phenomenon was observed in insulin signaling such as improved insulin sensitivity and glucose uptake. Further, when the authors induced animals to the ER stress, they observed an increase in TRB3 transcription levels concomitant with the losses in insulin signaling [23].

ER stress has been linked to insulin resistance in multiple tissues and is the major site in the cell for lipid and protein synthesis, folding, assembly, and trafficking, as well as cellular Ca^2+^ storage [24]. Another way how ER stress can induce the increase of TRB3 is through the increase of calcium in the cytosol. Obese people have higher calcium concentration in the cell due to the lipid-induced de-activation of the calcium pump [25] and to the opening of the IP3R ER calcium-channel. This is due to the activation of the glucagon receptor that is triggered by 2 processes: 1- The formation of IP3 by phospholipase C [26] and 2- The direct activation of the channel by PKA-mediated phosphorylation of IP3R [27]. This elevated calcium concentration will activate CaMKII, which will decrease p58^IPK^, induce ER stress, and activate PERK and ATF-4 pathways; this activation will in turn increase the synthesis of TRB3 [4].

In this regard, the TRB3 (*a mammalian homolog of Drosophila tribbles)* emerges as a negative regulator of the insulin signaling in several tissues, including the liver. Du et al. [28] found that in obese mice, there was an overexpression of the TRB3 protein that can be associated with a distal protein from the insulin pathway–the protein kinase B (Akt)– and can hence hinder Akt phosphorylation and its subsequent activation. Consequently, there was a reduction in insulin signaling on the insulin-sensitive tissues as well as other tissues such as the liver, triggering uncontrolled hepatic glucose production [28]. On the other hand, TRB3 inhibition reduces insulin resistance that is associated with obesity and helps control blood glucose levels [29].

## Insulin resistance state: the role of TRB3 in suppressing Akt activity

TRB3 is part of a new family of proteins and consists of three members (TRB1, TRB2 and TRB3), which are associated with numerous proteins including transcription factors and protein kinases (as well as E3 ubiquitin ligase). By interacting with different factors, the TRBs regulate many biological processes, including cell growth, differentiation, and metabolism [28]. TRB1 interacts with the mitogen-activated protein kinase (MAPK) modulating its kinase activity, resulting in muscle cell proliferation [30, 31]. TRB2 interacts with transcription factors C/EBP and is implicated in adipocyte differentiation [32]. TRB3 isoform, which has been the focus of several studies, emerges as a key player in in the regulation of insulin signaling through inhibition of Akt activity [28, 33, 34]. The effects of TRB3 in the organism remain unclear; for instance, TRB3 can have positive effects in the adipocytes because it can increase fatty acid oxidation [35]; conversely, TRB3—induced by ER stress—can have negative effects in the liver because it can decrease insulin signaling by binding to Akt and inhibiting it [36].

The role of TRB3 on Akt activity has been studied in different animal tissues. The amounts of TRB3 RNA and protein were increased in livers of db/db diabetic mice as compared to those in wild-type mice [28]. In both the skeletal muscle and the liver, TRB3 has been proposed to be a suppressor of Akt activity, mainly during fasting period and/or diabetic state [28, 37, 38]. The main role of hepatic TRB3 on Akt activity is to bind to Akt and then inhibit its phosphorylation at Thr^308^ and Ser^473^motifs, which are the critical motifs for Akt activation induced by growth factors and insulin [28]. Interestingly, when TRB3 was knockdown in the liver, Akt became active and the serum glucose levels went back to normal [37]. Conversely, when TRB3 was overexpressed in the liver, it promoted hyperglycemia and glucose intolerance [28],

Further, when TRB3 is overexpressed in the liver it promoted hyperglycemia and glucose intolerance, the same frame was showed in db/db mice that have high concentration of TRB3 in the liver [28]. Some authors have suggested that, by interfering with Akt activation, TRB3 contributes to insulin resistance in individuals with susceptibility to type 2 diabetes. Recently, Yu and colleagues [39] suggested that liver transcription of TRB3 levels may also be regulated by phosphoserine aminotransferase 1 (PSAT1), an enzyme that acts on the synthesis of the protein serine. The authors showed that PSAT1 inhibited the transcription of TRB3 in the liver and these results were accompanied by an increased activity of Akt and consequently a higher sensitivity to insulin and glucose uptake. Moreover, human polymorphism of TRB3 (Q84R) leads to an important inhibition of Akt activity and is related to the development of insulin resistance and an increased risk of cardiovascular disease [40].

In the liver, the expression of TRB3 is induced during fasting period in order to reduce insulin signaling at a time when the nutrient supply is not abundant, preventing the organism to reach a state of hypoglycemia [28, 33, 34]. After a meal, when the circulating insulin levels are increased, there is a 50 % reduction in the expression of TRB3 [38]. Elevated levels of TRB3 protein and the increased association of TRB3 with Akt are related to insulin resistance and, consequently, obesity and diabetes development [28, 33, 38].

In the liver of patients with DM2, TRB3 expression is increased independent of nutrients’ excess or hyperinsulinemia, leading to defects in insulin signaling and triggering a reduction in glycogen synthesis that is mediated by insulin as well as an increase in gluconeogenesis, which leads to hyperglycemia [28, 33, 34]. The negative effects of TRB3 block the activities stimulated by insulin, such as the inhibitory effect on hepatic glucose production and the inactivation of transcription factors for gluconeogenic enzymes (such as Foxo1 and PGC-1α) [28, 38].

Studies in obesity-animal models (either induced by a high fat diet or genetically modified) showed an increase in TRB3 expression and in the TRB3/Akt association in the skeletal muscle tissue and the liver, with a consequent increase in blood glucose concentration, which later on led to the development of insulin resistance and DM2 [28, 29, 41].

On the other hand, the inhibition of TRB3 activity increases the phosphorylation of Akt in response to insulin in the liver [28, 38]. Previous studies have shown that the absence of TRB3 is related to increased insulin sensitivity [28, 33]. Studies developed by our group, seeking to investigate the acute effect of physical exercise, showed a reduction in the expression of TRB3 protein in the skeletal muscle and liver of exercised animals [29, 41]. This phenomenon was associated with increased insulin sensitivity in animals induced to obesity by a high fat diet [29, 41]. These findings shed light on the importance of investigating the effect of, not only an acute period of exercise, but also physical training (chronic exercise) on TRB3 protein and its role in restoring insulin sensitivity in the liver tissue. Despite the need to define many other effects of physical exercise on intracellular signaling pathways, all these findings open new perspectives for understanding the effect of physical exercise on glycemic control and glucose production in the liver.

## The effect of exercise in the regulation of hepatic glucose production and the inhibitory effect of physical exercise on TRB3

Despite the beneficial effects of exercise on skeletal muscle and the increase in insulin sensitivity in animals and obese/diabetic patients, there is a lack of studies that demonstrate the role of physical exercise in regulating the insulin signaling pathway and the hepatic glucose production in the liver in these conditions.

In this regard, our research laboratory has sought to identify the effects of physical exercise on the liver tissue of obese and diabetic animals [29, 42–44]. The results have allowed a better understanding of the role of physical exercise in regulating some key proteins involved in glycogenolysis and gluconeogenesis in the liver.

A study that used a single session of aerobic exercise showed that exercise can decrease fasting hyperglycemia in obese mice induced by a high fat diet [42]. This effect on blood glucose was associated with a decrease in the association of Foxo1/PGC-1α inside the hepatocytes’ nucleus, consequently, it lowered the expression of the gluconeogenic enzymes PEPCK and G6Pase. There was also a reduction in hepatic glucose production, typically exaggerated under the condition of obesity and insulin resistance [42].

In the same direction, De Souza et al. [43] observed that a single session of physical exercise was able to decrease the expression of the nuclear factors Foxo1 and HNF-4α, both in genetically modified mice (*ob/ob*) and in Swiss obese mice induced by a high fat diet [43]. This cellular behavior was followed by a lower transcription of the gluconeogenic enzymes PEPCK and G6Pase and, consequently, a decrease in fasting glycemia in these two types of mice. Further, the obese animals subjected to physical exercise protocol had higher preservation and synthesis of hepatic glycogen [43]. Another study by Hoene et al. showed that after a single session of treadmill running, there was a rapid and pronounced increase of enzymes and transcription factors that regulated the hepatic metabolism such as G6Pase, pyruvate dehydrogenase kinase 4 (PDK4), IRS-2, PGC1- α, AMPK and Akt, which reinforces the idea that physical exercise might play a major role in TRB-3 activity [45].

To better understand the positive effect of physical exercise on insulin signaling in the liver of obese animals, Lima et al. investigated the effect of a single session of physical exercise on TRB3 protein levels and TRB3 association with Akt protein [29]. It was verified that the physical exercise reduced the expression of TRB3 and TRB3/Akt association. Consequently, such phenomenon was accompanied by an increase in Akt activity and its phosphorylation, leading to the inactivation of Foxo1. Associated with these adaptations, there was a reduction in the expression of the gluconeogenic enzyme PEPCK, both in *ob/ob* mice and in obese Swiss mice induced by a high fat diet [29]. Although these studies have demonstrated a significant effect of physical exercise on the TRB3 protein, they did not explain how this phenomenon happened.

To answer these questions, a study conducted by our research group described a new mechanism by which physical exercise could increase insulin signaling in the liver tissue while preserving Akt activity [44]. Aerobic physical training was able to increase the expression of endosomal adaptor protein expression (APPL1). This protein had the ability to inhibit the interaction between Akt and TRB3 in mice hepatocytes, which was accompanied by an increase in Akt activation in response to insulin stimulation [44].

Previous studies performed by Cheng et al. and Schenck et al. demonstrated that APPL1 regulated the activity of Akt [9, 46]. Both experiments with isolated cells, such as tissue analysis, showed that APPL1 competed directly with TRB3 in order to bind to Akt. Furthermore, overexpression of APPL1 could neutralize the inhibitory effect of TRB3 in the activation of Akt when stimulated by insulin and suppress the gluconeogenic program in the hepatocytes of obese mice [9].

Marinho et al. observed that aerobic training was able to increase the expression of APPl1 and Akt and the association between APPL1 and Akt protein in obese mice induced by a high fat diet. These results were confirmed by immunohistochemistry experiment, in which the obese mice submitted to physical training protocol showed higher levels of APPL1 in hepatic cells when compared to their sedentary pairs. Therefore, the increased expression of APPL1 resulted in an increased activation of Akt [44]. Moreover, it was also observed that physical training increased Foxo1 phosphorylation and decreased PGC-1α expression, reducing the association of these 2 proteins and consequently, the gluconeogenic program, by reducing the expression of PEPCK and G6Pase .

**Fig. 1:**
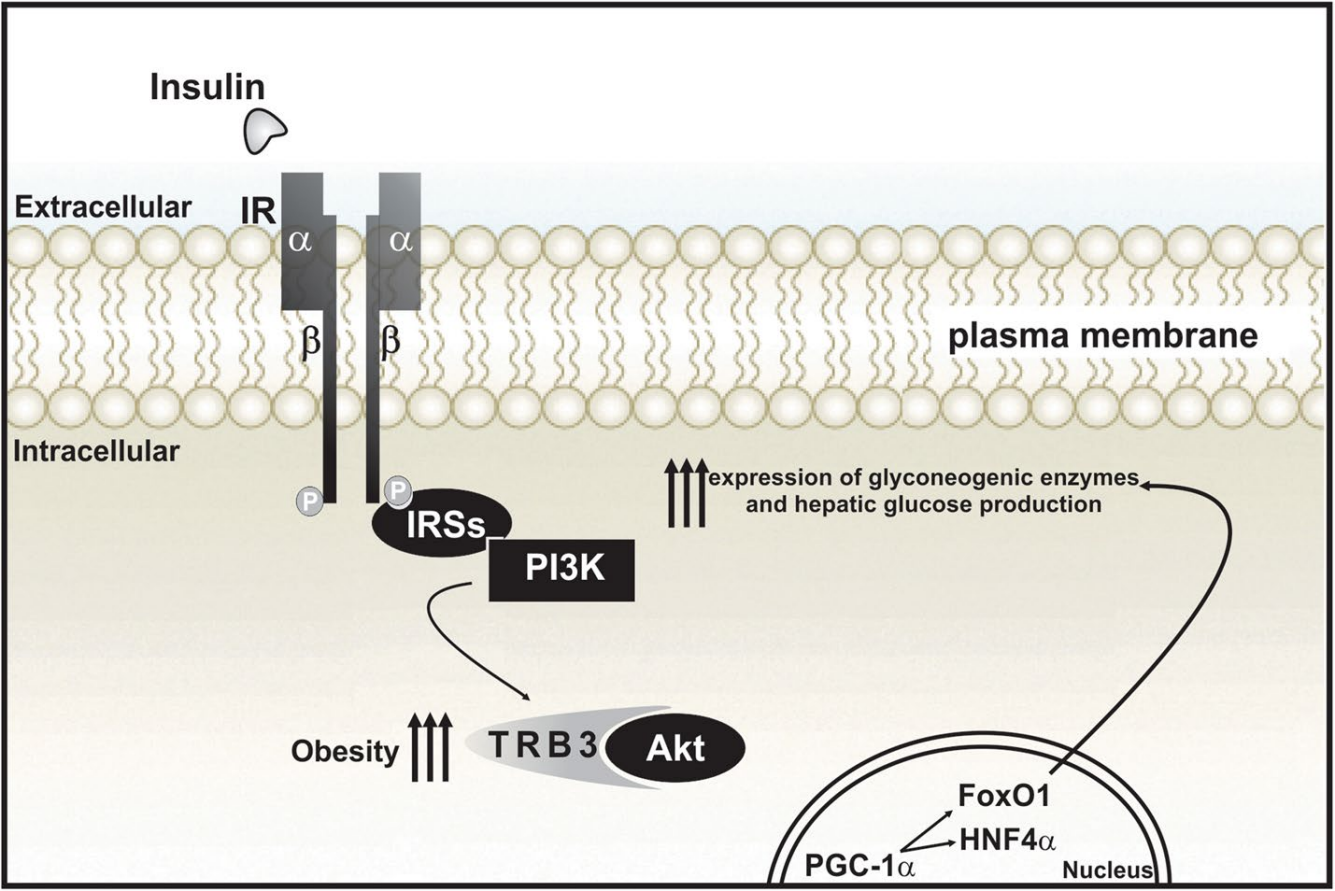
The increased expression of TRB3, induced by obesity or by the condition of insulin resistance, promotes a decrease in the phosphorylation of Akt and Foxo1, which enables the translocation of Foxo1 from the cytoplasm to the nucleus. Activated in the nucleus, the Foxo1 increases PGC-1α transcription and its association with this protein, starting the gluconeogenic program. As a consequence, there is an increase in the transcription of gluconeogenic enzymes, such as PEPCK and G6Pase in the liver resulting in hepatic glucose production

**Fig. 2:**
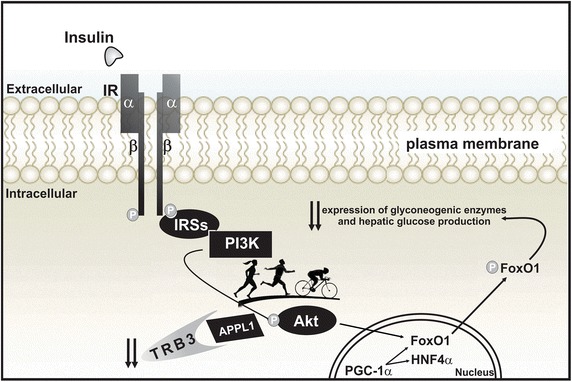
On the other hand, exercise markedly increases the expression of APPL1 and reduces the expression of TRB3, increasing hepatic insulin sensitivity and leading to the reduction of hyperglycemia due to a higher Akt phosphorylation. The activation of Akt allows the propagation of the insulin signal and the phosphorylation of Foxo1, which will exit the nucleus to the cytoplasm to decrease hepatic glucose production. These new findings allow us to better understand the molecular effects of physical exercise on the liver and its favorable effect on glycemic control and diabetes

In addition to this phenomenon, physical training increased the hepatic glycogen content due to increased phosphorylation of glycogen synthase enzyme kinase 3 (GSK3), leading to dephosphorylation and activation of glycogen syntheses (GS) [44]. In the skeletal muscle and the liver, insulin can stimulate the accumulation of glycogen by increasing its synthesis. This effect is obtained by dephosphorylation of the glycogen synthase enzyme (GS), where such dephosphorylation is only possible from the inhibition of GSK3. Under insulin stimulation, Akt phosphorylates GSK3 and inactivates it, which reduces the rate of GS phosphorylation, thus, increasing its activity. Therefore, in the presence of insulin, glucose may be stored as glycogen [44]. The potential mechanism of inhibition of gluconeogenesis by physical training through increasing APPL1 and inhibiting TRB3 is summarized in Figs. 1, 2.

## Conclusion

In summary, it can be concluded that physical exercise is an effective treatment to improve insulin sensitivity and contribute to glycemic control. Moreover, its effects go beyond the skeletal muscle, with specific adaptations on molecular pathways involved in hepatic gluconeogenesis regulation. Although future studies are needed to elucidate the effects of physical exercise on the liver, it seems clear that, when performed regularly, it can play an important role in preventing the development of insulin resistance and diabetes through the APPL1/TRB3/Akt pathway. Among the pronounced effects achieved by physical training, the inhibition of TRB3 in the liver could be highlighted, which leads to a decrease in gluconeogenesis in obese and diabetic animals. It is noteworthy that many of the observed effects were obtained independent of changes in body composition; this reinforces the importance of maintaining a physically active lifestyle to prevent insulin resistance and DM2.

## Abbreviations

TRB3: *Mammalian homolog of Drosophila tribbles*
DM2: Diabetes *mellitus* type 2
PEPCK: Phosphoenolpyruvate carboxykinase
G6Pase: Glucose-6-phosphatase
TLR4: Toll like receptor 4
IKKβ: Ikappakinase beta
JNK: c-Jun N-terminal kinase
MAPK: Mitogen-activated protein kinase
APPL1: Endosomal adaptor protein expression
GSK-3: Glycogen synthase enzyme kinase 3
GS: Glycogen synthase
